# New York Odontological Society

**Published:** 1885-07

**Authors:** 


### NEW YORK ODONTOLOGICAL SOCIETY.

The regular monthly meeting of this society was held at the residence of Dr. Geo. S. Allan; President Jarvie in the chair.

*Dr. Bodecker*—Presented samples of copper amalgam which he had received from Germany, and which he considered of much value. He had employed this amalgam for lining the cavity walls before introducing other amalgams in general use.

*Dr. A. H. Brockway*—Asked what peculiar advantages the copper amalgam possessed over other amalgams ?

*Dr. Bodecker*—Thought copper amalgam made a more compact filling, but his objection to filling cavities entirely with this material, was its liability to discolor in the mouth, as it is too apt to do. Dr. B. was asked if a perfect union took place between the copper and other amalgams when placed side by side in the same cavity. In reply, Dr. B. thought it possible, but had not used it a sufficient length of time to speak positively on that point.

*Dr. E. A. Bogue*—Stated that no union would take place in such cases, and that where such a plug is removed from its cavity the silver amalgam would scale from the copper filling.

The matter of "systemic effects" produced by copper amalgam was mentioned.

*Dr. Brockway*—Had seen large stoppings of this material, that had been in patients' mouths for a great length of time, showing no ill effect and causing no complaints whatever.

*Dr. W. H. Waite*, of Liverpool, England, who was present, stated that in England it was a common event to see copper amalgam stoppings in patients' teeth, and he had never heard of any complaint regarding disagreeable taste from its presence, nor had he ever heard of any ill effect to the system caused by them. He had not used it much in his own practice, as he preferred silver amalgam.

Samples of copper amalgam were presented to members of the society by Dr. Bodecker, for trial or experiment.

*Dr. Brockway*—Referred to a paper read at the previous meeting by Dr. Clowes, upon "Separation," which was not then discussed, and he desired the author to explain his operations or methods of separation, that the members present might better understand them.

*Dr. Clowes*—Expressed himself as greatly surprised to find that his paper had awakened this degree of interest, and that he should be called upon for further explanation, especially as his efforts in this direction were usually received with incredulous smiles. He, however, explained his practice of separating, which, although it was by some pronounced painful and barbarous, he performed even upon the teeth of children, who went through the operation as if they were enjoying a sort of frolic. Being asked by Dr. Brockway what proportion of his patients had their teeth thus separated, Dr. Clowes replied, "Almost every one." He further stated that it was a well known and simple fact, that where the teeth naturally stood apart there was no decay, but when so close together as to hold food they were always in danger of decay. When a young operator he *thought* separation good practice; now, as an old operator, he *knew* it to be so.

A paper sent by Dr. Beers, of Montreal (who was detained by sickness), was read by the Secretary.

Dr. Waite, of Liverpool, was formally introduced to the Society, and read an exceedingly interesting paper on the subject of "Dental Education in England and in the United States." He premised by stating that the course of study and examination for the degree of L. D. S. in England was as rigid and thoroughly conducted as for the degree of M. D. The English system, as to *theory*, in examinations, he considered excellent, but they greatly lacked *practical* demonstrations in operations before examiners, which American colleges insisted upon. He congratulated the profession upon the continued advancement made in the educational departments, and especially upon the introduction of preliminary examinations. The doctor did not believe in the system of having the examinations conducted by the professors, but thought, as in England, they should have an examining board appointed, whose duty it should be to visit the different colleges and examine into the qualifications of students.

Dr. Waite then exhibited to the society some specimens of prehistoric dentistry, consisting of artificial teeth attached to gold bands or collars, made to fit the necks of the natural organs, the same that were described by him in the April number of this Journal. These specimens were obtained by Dr. Waite as a loan from one of the English museums. The specimens were much like those represented in the January number of the INDEPENDENT PRACTITIONER, in the contribution of Dr. Van Marter, of Rome.

The thanks of the society were tendered to Dr. Waite for his paper, and for his trouble in obtaining and exhibiting the very interesting specimens.

